# Supplementary material for: Overexpression of SgGH3.1 from Fine-Stem Stylo (Stylosanthes guianensis var. intermedia) Enhances Chilling and Cold Tolerance in Arabidopsis thaliana
Source: Genes (Basel). 2021 Aug 31;12(9):1367. doi: 10.3390/genes12091367 (PMC8469043; doi:10.3390/genes12091367)
Supplement: Supplementary file 1 [file genes-12-01367-s001.zip › genes-1350142-supplementary.pdf]

genes

Table S1 Primer pairs used in PCR and Realtime PCR

Primer ID	Primer sequence (5'-3')	Product size (bp)	GenBank accession number of target gene
SgGH3.1-pBA-F	TCTAGAATGGCCATTGGTTCTGTGTTCT		
SgGH3.1-pBA-R	GGATCCTCAACAACGCCGTTCCGGGGT		
SgGH3.1-qRT-F	ACTACCTCGTCGGGATTGTG		
SgGH3.1-qRT-R	CGTCGAACTCCTTCAAGAGC		
AtCBF1-qRTF	GCATGTCTCAACTTCGCTGA	161	NM_118681.4
AtCBF1-qRTR	ATCGTCTCCTCCATGTCCAG		
AtCBF2-qRTF	TGACGTGTCCTTATGGAGCTA	170	NM_118679.2
AtCBF2-qRTR	CTGCACTCAAAAACATTTGCA		
AtCBF3-qRTF	GATGACGACGTATCGTTATGGA	144	NM_118680.2
AtCBF3-qRTR	TACACTCGTTTCTCAGTTTTACAAAC		
AtACTIN2_qRTF	TAACAGGGAGAAGATGACTCAGATCA	199	NM_121018.4
AtACTIN2_qRTR	AAGATCAAGACGAAGGATAGCATGAG		

genes MDPI

Table S2 The information of the 20 *Arabidopsis thaliana* GH3 proteins used for phylogenetic tree construction

Protein ID	Accession	Description
AtGH3-1	AAC61292.1	putative auxin-regulated protein [Arabidopsis thaliana]
AtGH3-10	AAD14468.1	putative GH3-like protein [Arabidopsis thaliana]
AtGH3-11/FIN219	AAD23040.2	putative auxin-responsive protein [Arabidopsis thaliana]
AtGH3-12	CAB86639.1	auxin-responsive-like protein [Arabidopsis thaliana]
AtGH3-13	CAB86642.1	auxin-responsive-like protein [Arabidopsis thaliana]
AtGH3-14	CAB87143.1	auxin reponsive-like protein [Arabidopsis thaliana]
AtGH3-15	CAB87144.1	auxin reponsive-like protein [Arabidopsis thaliana]
AtGH3-16	CAB87145.1	auxin reponsive-like protein [Arabidopsis thaliana]
AtGH3-17	AAF98442.1	Unknown protein [Arabidopsis thaliana]
AtGH3-18	AAG60120.1	Nt-gh3 deduced protein, putative [Arabidopsis thaliana]
AtGH3-19	AAG60122.1	Nt-gh3 deduced protein, putative [Arabidopsis thaliana]
AtGH3-2	CAB38206.1	auxin-responsive GH3-like protein [Arabidopsis thaliana]
AtGH3-20	AAG60118.1	auxin-regulated protein, putative [Arabidopsis thaliana]
AtGH3-3	AAB87114.1	unknown protein [Arabidopsis thaliana]
AtGH3-4	AAF79776.1	T30E16.2 [Arabidopsis thaliana]
AtGH3-5	CAA19720.1	GH3 like protein [Arabidopsis thaliana]
AtGH3-6	BAA97524.1	auxin-responsive-like protein [Arabidopsis thaliana]
AtGH3-7	AAC00604.1	highly similar to auxin-regulated protein GH3, gp|X60033|18591 [Arabidopsis thaliana]
AtGH3-8	BAB08663.1	auxin-responsive GH3-like protein [Arabidopsis thaliana]
AtGH3-9	AAC63630.1	putative auxin-responsive protein [Arabidopsis thaliana]

genes

MDPI

Table S3 The information of the 14 legume GH3.1 proteins used for multiple sequence alignment and phylogenetic tree construction

Accession	Description	Scientific Name	Common Name	Taxid
XP_025627298.1	probable indole-3-acetic acid-amido synthetase GH3.1 [Arachis hypogaea]	*Arachis hypogaea*	peanut	3818
XP_015956128.1	probable indole-3-acetic acid-amido synthetase GH3.1 [Arachis duranensis]	*Arachis duranensis*	NA	130453
XP_016207718.1	probable indole-3-acetic acid-amido synthetase GH3.1 [Arachis ipaensis]	*Arachis ipaensis*	NA	130454
TKY70621.1	indole-3-acetic acid-amido synthetase GH3.1 [Spatholobus suberectus]	*Spatholobus suberectus*	NA	455371
KAF7828658.1	putative indole-3-acetic acid-amido synthetase GH3.1 [Senna tora]	*Senna tora*	NA	362788
XP_027361377.1	probable indole-3-acetic acid-amido synthetase GH3.1 [Abrus precatorius]	*Abrus precatorius*	Indian licorice	3816
KAG4973189.1	hypothetical protein JHK87_030010 [Glycine soja]	*Glycine soja*	NA	3848
XP_003539320.1	probable indole-3-acetic acid-amido synthetase GH3.1 [Glycine max]	*Glycine max*	soybean	3847
XP_028766877.1	probable indole-3-acetic acid-amido synthetase GH3.1 [Prosopis alba]	*Prosopis alba*	NA	207710
KAF1872423.1	hypothetical protein Lal_00016721 [Lupinus albus]	*Lupinus albus*	white lupine	3870
XP_020230361.1	probable indole-3-acetic acid-amido synthetase GH3.1 [Cajanus cajan]	*Cajanus cajan*	pigeon pea	3821
XP_017412459.1	PREDICTED: probable indole-3-acetic acid-amido synthetase GH3.1 [Vigna angularis]	*Vigna angularis*	adzuki bean	3914
RDY08267.1	putative indole-3-acetic acid-amido synthetase GH3.1 [Mucuna pruriens]	*Mucuna pruriens*	NA	157652
KAE9614265.1	putative GH3 family protein [Lupinus albus]	*Lupinus albus*	white lupine	3870
AWN02115.1	indole-3-acetic acid-amido synthetase [Trifolium repens]	*Trifolium repens*	white clover	3899
XP_028244106.1	probable indole-3-acetic acid-amido synthetase GH3.1 [Glycine soja]	*Glycine soja*	NA	3848
XP_003611653.1	probable indole-3-acetic acid-amido synthetase GH3.1 [Medicago truncatula]	*Medicago truncatula*	barrel medic	3880

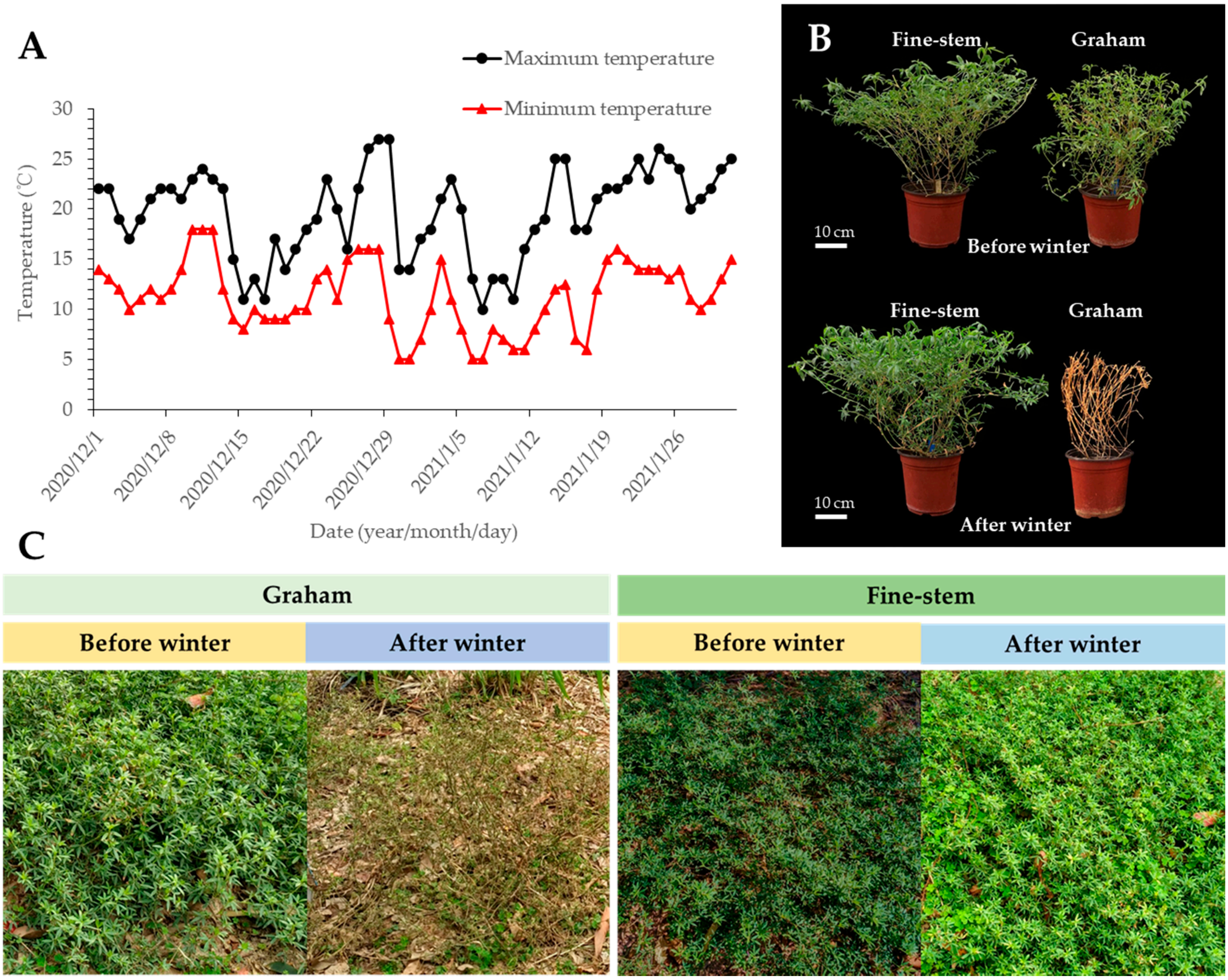

Figure S1. The growth of fine-stem stylo (*Stylosanthes guianensis* var. *intermedia*) and *S guianensis* cv. Graham before and after winter in Guangzhou, China. (**A**) The temperature fluctuation through the winter of year 2020-2021. (**B**) The growth of fine-stem and 'Graham' stylo in pot before and after winter. (**C**) The growth of fine-stem and 'Graham' stylo in field before and after winter.

genes

MDPI

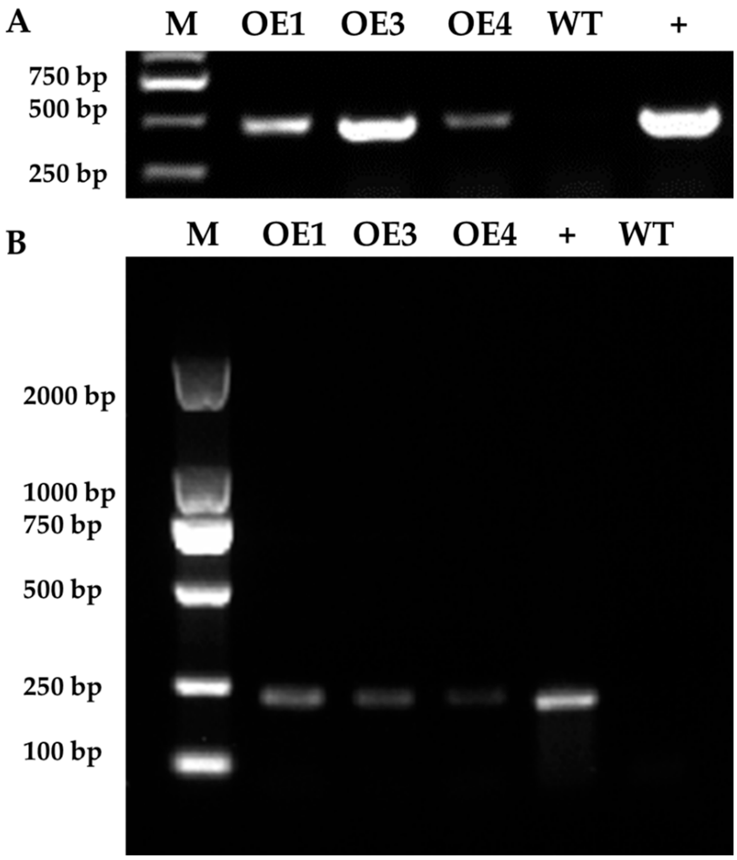

Figure S2. PCR amplification of *bar* (**A**) and *SgGH3.1* (**B**) genes from wild-type (WT) and *SgGH3.1* overexpressing (OE1, 3 and 4) *Arabidopsis thaliana*. M: 2000 bp DNA marker; +: recombinant plasmid pBA002-*SgGH3.1*.

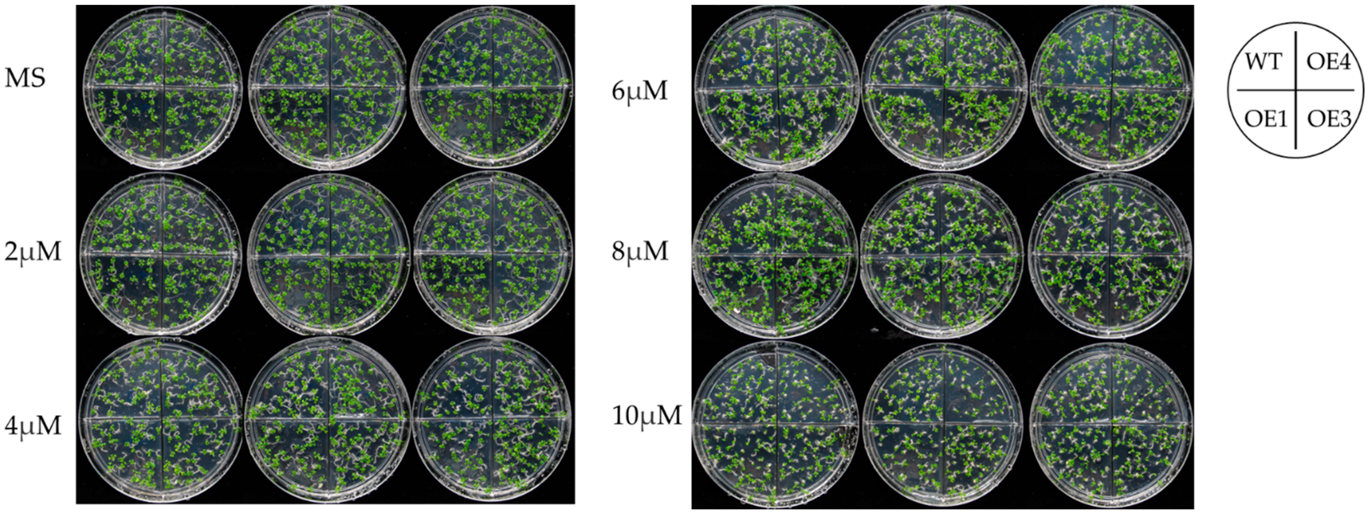

Figure S3. The growth of wild-type (WT) and SgGH3.1 overexpressing (OE1, 3 and 4) *Arabidopsis thaliana* after 18 days' cultivation in MS medium supplemented with different concentrations of IAA.
